# The Economics of Municipal Charity

**Published:** 1895-12

**Authors:** 


A Monthly Review of Medicine and Surgery.

EDITORS:

THOMAS LOTHROP, M. D. - - WM. WARREN POTTER, M. D.

All communications, whether of a literary or business nature, should be addressed to the managing editor: 284 FRANKLIN STREET, BUFFALO, N. Y.

VOL. XXXV. DECEMBER, 1895. No. 5.

### THE ECONOMICS OF MUNICIPAL CHARITY.

THE season of the year has again arrived when the question of the care of the poor stands out for consideration with accentuated prominence. While a great city like Buffalo always has at hand a goodly number of individuals whose squalid environment and helplessness from age and sickness constantly appeals to the sympathy of a liberal-hearted people, yet it is in the cold weather that these and other impoverished and unemployed individuals present the greatest claim to public and private charity.

From December to May, in Buffalo, there is less opportunity for employment than at other seasons of the year. The traffic of the great lakes and canals is suspended during this period, and many other collateral business interests, as a consequence, come to a standstill. So, between the sick and aged poor, whom we always have with us, and the influx of laborers suddenly made idle by the close of navigation and a suspension of its attendant industries, there is a problem presented for solution which may well attract the attention of philanthropists, municipal officers and sanitarians.

To deal successfully with this important question in political economy there must be a concert of action on the part of these three groups. Municipal officers, represented in the mayor and common council and the board of public works, must be in accord with the health commissioner and his departmental staff, and these, in turn, must be aided and supported by philanthropic, charitable and large-hearted citizens. With a unity of action among and between these several elements, the suffering and mortality of this great city growing out of its severe winter climate, as directly related to the poor, may be reduced to a minimum.

It is our purpose, at this time, to discuss this question from the standpoint of preventive medicine, which is now and from henceforth will continue to be the great province of physicians. The state has assumed control of medical practice, insisting that physicians must be better educated than formerly and that they shall not be permitted to exercise their functions except under a license which issues after due and adequate examination. By this action the state commissions the doctor as one of its agents to minister unto disease, and it specially charges these, its agents, to go forth into every quarter of the commonwealth and seek to arrest sickness through the application of the most scientific and efficient methods of preventive medicine yet known.

One of the highest functions of prevention may be exercised through channels that relate to the health of the poor. If these unfortunate persons can be kept well they may be made, at least in part, self-supporting. To prevent disease among this class of citizens, nutrition, warmth and cleanliness must receive adequate attention. Starvation, cold and filth are very sure breeders of ill-health. If the poor, through neglect, are allowed to become sick, they cease to be producers and at once become a public charge, and hence a double expense. They then must be cared for either through public or private charity; there is no escape from this mandate. Humanity everywhere has no instinct that does not prompt a tender care of the sick.

The question that we would present for the consideration of the municipality, officially and collectively, is, How can this most important problem be adequately and economically dealt with? Great financial enterprises are absorbing wealthy and public-spirited citizens, and the municipality in its corporate capacity is asked to lend a helping hand, either by direction or indirection, to many of these worthy schemes for the advancement of public and individual interests.

Greater Buffalo is attracting the attention of the business world. Now is a time when Buffalo is on trial before the most critical and astute court in the universe—namely, public opinion. Let it go forth that it has an unassailable system for the proper care of its poor. Let it be understood that we recognise fuel, food, clothing, cleanliness and employment as great sanitary allies and that we deal them out with a liberal hand, yet not recklessly, as preventive measures in the health economics of this great city.

It is economical for the department of public works to employ with a liberal hand on our streets during the winter months, to the end that they may be kept free from snow and ice blockades. The railroads throw the snow up into impassable barriers that should be at once carted away from the principal business streets. And let it especially be remembered that for every shovel employed the city is giving an object lesson in preventive medicine, which means municipal economy.

